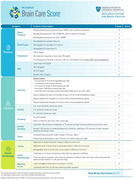# The Brain Care Score and its Associations with Dementia and Stroke Incidence in the UK Biobank and REGARDS cohorts

**DOI:** 10.1002/alz70860_101096

**Published:** 2025-12-23

**Authors:** Sanjula D Singh

**Affiliations:** ^1^ Harvard Medical School, Boston, MA, USA

## Abstract

**Background:**

The Brain Care Score (BCS) was developed to assist individuals to make health‐related behaviors changes associated with a lower incidence of dementia and stroke by addressing 12 modifiable risk factors (*Figure 1*). This study evaluated the associations between the BCS and incident dementia and stroke across two different cohorts.

**Method:**

Using a two‐stage discovery and replication design, we analyzed data from the UK Biobank (UKB) and the REasons for Geographic and Racial Differences in Stroke (REGARDS) cohorts from the United Kingdom and United States, respectively. The BCS was categorized into three categories: low (Q1), intermediate (Q2–4), and high (Q5). A higher BCS represents improved modifiable risk factor control for dementia and stroke incidence. We assessed stroke and dementia/cognitive impairment incidence, analyzed using multivariable Cox proportional hazards models, adjusted for sex and age – presenting Hazard Ratios (HRs) and 95% Confidence Intervals (95%CI).

**Result:**

Amongst 391,399 UKB participants (mean age: 57 years; 54% female, 94% self‐reported white ethnicity), 8,376 strokes (2.1%) and 6,543 dementia cases (1.7%) occurred during 13.2 years of median follow‐up. Compared to low BCS, intermediate and high BCS was associated with lower incidence of stroke by 32% (HR:0.68; 95%CI:0.65–0.71) and 45% (HR:0.55; 95%CI:0.51–0.59), respectively, and lower incidence of dementia by 14% (HR:0.86; 95%CI:0.81–0.91) and 13% (HR:0.87; 95%CI:0.80–0.94), respectively. Amongst 10,635 REGARDS participants (mean age: 63 years; 58% female, 70% self‐reported white ethnicity), 660 strokes (6.2%) and 533 cognitive impairment cases (5.0%) occurred during 15.1 years of follow‐up. Compared to low BCS, intermediate and high BCS was associated with a lower stroke incidence by 34% (HR:0.66; 95%CI:0.55–0.78) and 38% (HR:0.62; 95%CI:0.50–0.78), respectively, and cognitive impairment incidence by 49% (HR:0.51; 95%CI:0.42–0.62) and 51% (HR:0.49; 95%CI:0.39–0.63).

**Conclusion:**

The BCS has clinically relevant and statistically associations with dementia and stroke incidence in participants from two ethnically diverse cohorts: the UKB and REGARDS. Use of the BCS to highlight incidence of dementia and stroke in primary care or other clinical settings may help stimulate conversations around brain health and promote risk factor modification to prevent or delay dementia or stroke